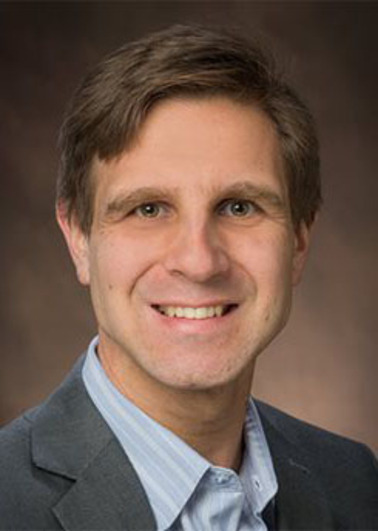# Dynamic Bonds: Adaptable Timescales for Responsive Materials

**DOI:** 10.1002/anie.202206938

**Published:** 2022-11-02

**Authors:** Shiwanka V. Wanasinghe, Obed J. Dodo, Dominik Konkolewicz

**Affiliations:** ^1^ Department of Chemistry and Biochemistry Miami University 651 East High Street Oxford OH 45056 USA

**Keywords:** Cross-Linking, Dynamic Bonds, Dynamic Polymers, Responsive Materials, Tuning Dynamic Bonds

## Abstract

Dynamic bonds introduce unique properties such as self‐healing, recyclability, shape memory, and malleability to polymers. Significant efforts have been made to synthesize a variety of dynamic linkers, creating a diverse library of materials. In addition to the development of new dynamic chemistries, fine‐tuning of dynamic bonds has emerged as a technique to modulate dynamic properties. This Review highlights approaches for controlling the timescales of dynamic bonds in polymers. Particularly, eight dynamic bonds are considered, including urea/urethanes, boronic esters, Thiol–Michael exchange, Diels–Alder adducts, transesterification, imine bonds, coordination bonds, and hydrogen bonding. This Review emphasizes how structural modifications and external factors have been used as tools to tune the dynamic character of materials. Finally, this Review proposes strategies for tailoring the timescales of dynamic bonds in polymer materials through both kinetic effects and modulating bond thermodynamics.

## Graphical abstract

For dynamic polymer materials to respond to their environment and be tailored towards an application, the timescale of bond exchange must be tuned. Both chemical structural modifications and external factors such as temperature, catalyst, and matrix can be used to fine‐tune the timescales of exchange. This Review focuses on the tools available to modulate the timescales of dynamic bonds in polymeric materials.

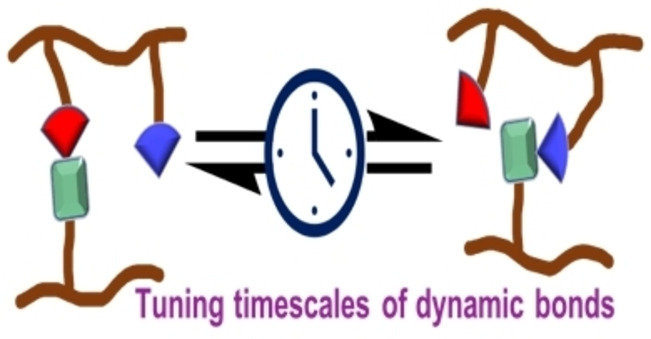

## 1 Introduction

Dynamic bonds and interactions have impacted the fields of organic chemistry, materials, and nanoscience. By enabling linkers to exchange, dynamic chemistry adds responsiveness and adaptability to otherwise static materials.^[1]^ Dynamic bonds have meaningful rates of bond exchange while maintaining a balanced bond density, consistent with the concept that at equilibrium, the rates of forward and reverse reactions are equal. However, in response to applied forces or stress or damage to the material, dynamic bonds exchange to restore an equilibrium configuration.

The exchange of dynamic bonds can either be autonomous under ambient conditions, or driven by external stimuli such as heat, light, pH, etc. Dynamic bonds can be subcategorized into two main classes based on their bond type: dynamic noncovalent bonds (DNBs) and dynamic covalent bonds (DCBs).[^2^, ^3^, ^4^, ^5^, ^6^] DNBs are generally based on rapidly exchanging supramolecular interactions such as hydrogen bonding,[^7^, ^8^, ^9^, ^10^] π–π stacking,[^11^, ^12^, ^13^] host–guest interactions,[^14^, ^15^, ^16^, ^17^, ^18^] ionic interactions,[^19^, ^20^] and metal coordination.[^21^, ^22^, ^23^] DCBs are typically stimuli‐responsive dynamic covalent reactions such as Diels–Alder cycloaddition,[^24^, ^25^, ^26^] Thiol–Michael reactions,[^27^, ^28^, ^29^, ^30^] urea exchange,[^31^, ^32^, ^33^] imines,[^32^, ^34^, ^35^, ^36^] boronic esters and boronates, etc.[^37^, ^38^, ^39^, ^40^, ^41^, ^42^, ^43^] Each dynamic interaction has a characteristic timescale of exchange. The timescale of dynamic bond exchange is correlated with the timescale at which the material will respond. Although there are general trends, e.g., DNBs tend to exchange faster than DCBs under ambient conditions, it is possible to modulate a given dynamic bond to broaden its timescales of exchange.^[44]^

**Scheme 1:**
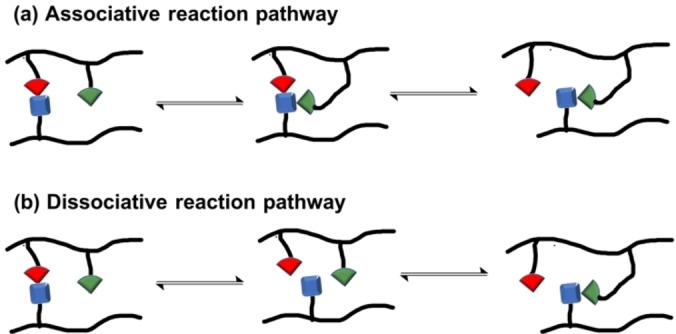
Dynamic bond exchange pathways: a) Associative reaction mechanism and b) dissociative reaction mechanism.

Dynamic bonds exchange either through associative or dissociative pathways. Dissociative pathways undergo a bond breaking followed by bond reformation while the associative pathway has near simultaneous bond breakage and a bond formation (Scheme 1).^[44]^ However, a recent report suggests that the scaling of characteristic exchange timescales follows an Arrhenius relationship in both pathways.^[45]^ Incorporation of reversible bonds into polymer networks has been of great interest to the scientific community due to resulting tailorable mechanical properties such as malleability, self‐healing, shape memory, stress relaxation, and recyclability.[^29^, ^30^, ^46^, ^47^, ^48^, ^49^, ^50^, ^51^, ^52^] Due to the tremendous properties of dynamic materials, numerous potential applications are available including medicinal applications,[^53^, ^54^, ^55^] electrically conductive materials,[^56^, ^57^, ^58^, ^59^] soft robotics,[^60^, ^61^] fiber‐reinforced composites,^[62]^ energy storage,^[63]^ and electronic skins.^[64]^

Recent reviews have highlighted adaptable polymer networks using DCBs[^36^, ^49^, ^65^] and DNBs.[^44^, ^66^, ^67^, ^68^] Although there are many discussions on dynamic chemistries, control over their dynamic characteristics and timescales in bulk polymer materials has received less attention. Bond exchange rates can be tuned from essentially static to extremely fast and on the order of seconds. This exchange time depends on the structure of the linker and the presence or absence of applied stimulus.

Fine‐tuning the bond exchange timescales will impact the material's responsiveness. Several strategies have been developed to adjust the kinetics of these dynamic bonds over the past years. This Review focuses on tuning of dynamic bonds where the bond exchange kinetics correlate with dynamic characters in polymer networks. The recent progress made in the area of fine‐tuning dynamic bonds and how it affects material properties will be explored, with emphasis on the timescale of bond exchange. This Review will address 8 types of dynamic bonds: urea/urethanes, boronic esters, Thiol–Michael exchange, Diels–Alder reactions, transesterification, imine bonds, coordination bonds, and hydrogen bonds. Stress relaxation, self‐healing properties, toughness, and strength of polymer materials will be used as metrics to capture the impact of bond exchange rates.

## 2 Tuning Timescales of Exchange and the Corresponding Impact on Materials

Tuning dynamic bond exchange rates can lead to adaptable materials. For instance, hydrolysable polymers are used in many biomedical applications and eco‐friendly packaging materials that need to maintain structure for a period of time as well as full degradation of the material after the function is completed.^[69]^ In addition to degradability and self‐healing ability, stress relaxation rates of dynamic materials can be controlled by tuning the kinetics of dynamic bond exchange, which influences the recyclability of materials.^[70]^ Rapid exchange of dynamic bonds can improve the self‐healing and stress relaxation of polymer networks, although long‐term stability, such as creep resistance, is improved with slower bond exchange.

Bond dissociation energy is often correlated to the properties of polymers. Lowering bond energies can make bonds more reversible under mild conditions, especially if bond exchange occurs through a dissociative mechanism. However, appropriate balance of bond energies should be maintained since weak bonds can lead to creep‐susceptible polymers, which can limit performance under load.^[71]^ There are several modifications that can be used to control the kinetics of dynamic exchange. Electronic effects and steric effects are the most popular strategies used to modulate the dynamics of materials.

**Scheme 2:**
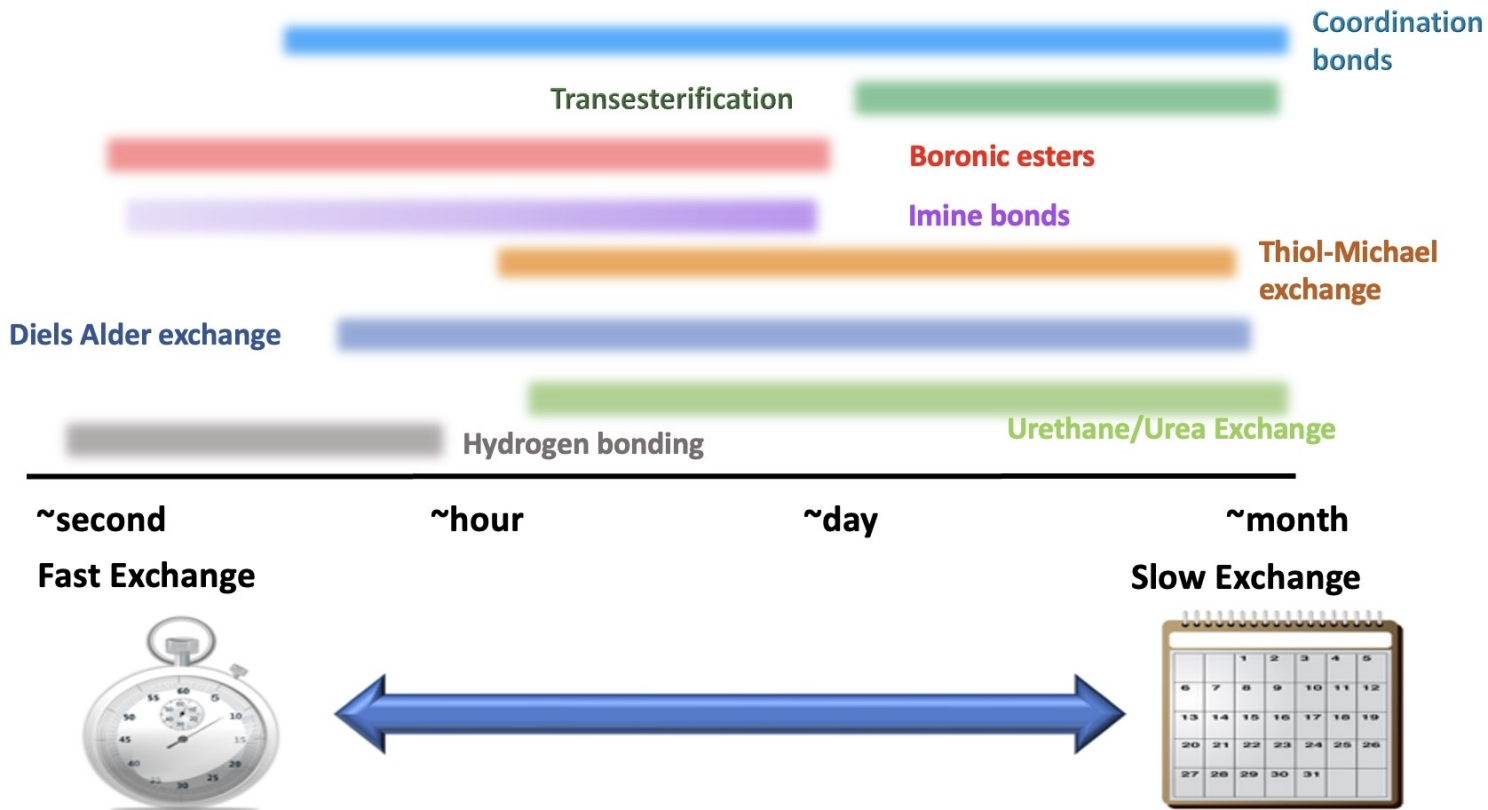
Relative timescales of dynamic bond exchanges at ambient and near‐ambient conditions.

In addition to structural modifications, external factors such as temperature and matrix effects have been identified as potential tuning parameters.[^72^, ^73^] As seen in Scheme 2, the exchange timescales of dynamic bonds can not only be modulated by the fundamental choice of dynamic chemistry, but can also be fine‐tuned within a class of dynamic bonds by the steric and electronic effects as well as applied stimuli. Scheme 2 gives common ranges of exchange timescales for various bonds although systems may be tuned outside this approximate range.

## 3 Urethanes/Urea

**Scheme 3:**
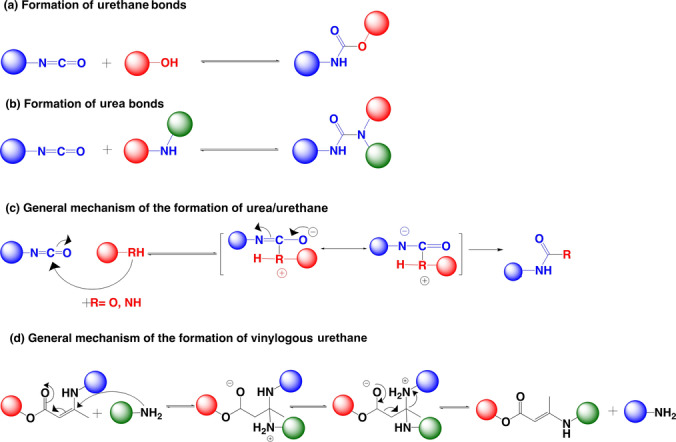
Basic reaction schemes for the formation of a) urethane and b) urea bonds. c) General mechanism of the reaction between an isocyanate and a nucleophile. d) General mechanism of vinylogous urethane exchange.

The electrophilic carbon of an isocyanate group readily reacts with active nucleophiles such as thiols, alcohols, and amines. Urethane and urea bonds are formed by the reaction of an isocyanate group with an alcohol and amine, respectively^[73]^ (Scheme 3a–c).^[74]^ The reversibility of the urethane/urea bond has been used to synthesize materials with interesting applications such as adhesives, sealants, coatings, and fibers.[^75^, ^76^] From the perspective of reversibility, dissociation of urethane/urea bonds is proposed to occur through a dissociative bond exchange mechanism, although urethanes can also follow the associative pathway with free hydroxy groups.^[2]^ The equilibrium between reactants and products of this reaction is highly dependent on the electronic effects of substituents, solvent effect, dielectric constant, polarity, hydrogen‐bonding ability, temperature, and other matrix effects.^[73]^ Therefore, reversibility can be controlled by varying the abovementioned factors guided by appropriate small‐molecule‐based studies.

The carbon–nitrogen linkages in urea are extremely stable due to conjugation of the lone pair of the nitrogen atom and the π‐electrons of the carbonyl group.^[77]^ It requires extreme conditions such as high temperature, high pH or very low pH, or other additives to reverse the reaction.[^77^, ^78^] This restricts the applications of unmodified urea bonds in polymeric materials, owing to their lack of recyclability, self‐healing, and processability. To overcome the slow dynamics of ureas, studies have modified the urea group to facilitate exchange. Weakening the carbonyl–nitrogen interaction of urea is one strategy to increase the dynamic character of urea bonds. Incorporation of bulky substituents to the nitrogen atom weakens the urea by disturbing the geometrical co‐planarity of the amide unit. This reduces the conjugation between the nitrogen atom and the carbonyl group making it vulnerable to amidolysis even under relatively mild conditions.^[79]^ Isocyanates are used to prepare urea‐based polymers due to the ease of handling the dissociated carbonyl group under ambient conditions, while enabling possible urea bond exchange with amines.^[77]^

The dynamic character of urea bonds can be modulated by changing the forward/reverse rate coefficients (*k*_1_ and *k*_−1_) and the equilibrium constant (*K*_eq_). Both *k*_1_ and *k*_−1_ should be relatively large to enable bond exchange, and it is important that *K*_eq_ be sufficiently large to shift the equilibrium towards the formation of chains with high degree of polymerization or high cross‐link density.[^69^, ^77^] These parameters can be controlled by changing the bulkiness of substituents to the nitrogen atom of urea or urethane bonds.

**Figure 1:**
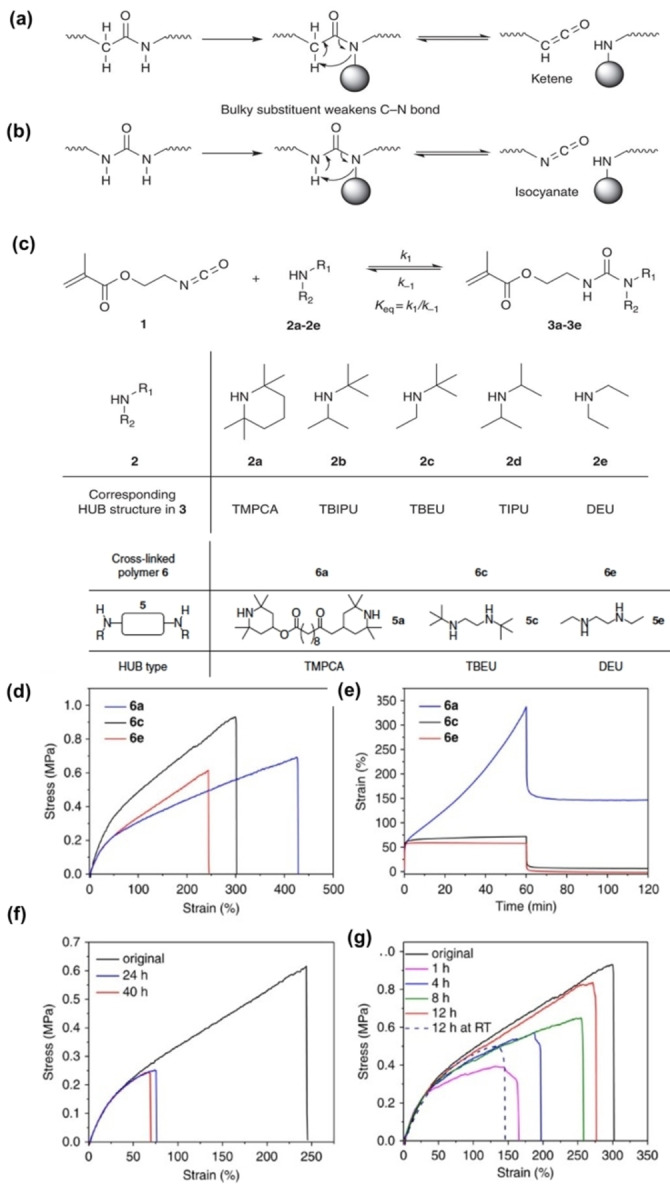
Dissociation of carboxylate/amine bonds bearing a bulky N‐substituent. a) Hindered amide bond dissociates to unstable ketene intermediate. b) HUB dissociates to isocyanate. c) Equilibrium between isocyanate **1**, bulky amines [**2 a**–**e**] and corresponding ureas [**3 a**–**e**], the chemical structures of bulky amines [**2 a**–**e**] and the urea **3 a**–**e** bearing the corresponding HUB (TMPCA), (TBIPU), (TBEU), (DIPU), and (DEU). Mechanical characterization of HUB‐based cross‐linked poly(urethane‐urea). Diamines **5 a**, **5 c**, and **5 e** were used to form the corresponding cross‐linked networks (TMPCA, TBEU, and DEU) **6 a**, **6 c**, and **6 e**, respectively. d) Stress–strain curves of **6 a**, **6 c**, and **6 e**, which are the corresponding HUB motifs (TMPCA, TBEU, and DEU) in the desired network polymers, respectively. e) Creep recovery of **6 a**, **6 c**, and **6 e** with initial strain of 50 %. f) Inefficient recovery of the breaking strain of **6 e** after a long period of healing. g) Recovery of breaking strain of sample **6 c** under various healing conditions. The healing at room temperature (RT) is less efficient (dotted line) (Adapted from the ref. [77]).

For example, Ying et al. designed polyureas and poly(urethane—urea)s with catalyst‐free reversibility by incorporating sterically hindered dynamic urea bonds (Figure 1a—c).^[77]^ Dynamicity increased with the bulkiness of the substituent attached to the nitrogen atom, where the most hindered amide bond showed high dynamicity, high *k*_−1_, while the least hindered amide bond showed less dynamicity, low *k*_−1_. However, extremely hindered urea bonds gave very low *K*_eq_ values. Materials with these extremely hindered urea bonds act as weakly cross‐linked networks that yielded upon stretching. Hence it is important to tune the timescales within this frame of appropriate *K*_eq_ and *k*_−1_ for targeted applications. As expected, fast self‐healing was achieved with moderate bulkiness and poor self‐healing was seen with low bulkiness. Hindered urea bonds (HUBs) indicate that dynamic properties are impacted by steric effects in urea bonds (Figure 1e–g). Hence, modulating the bulkiness of substituents is an effective tuning strategy in urea‐based polymers.

**Figure 2:**
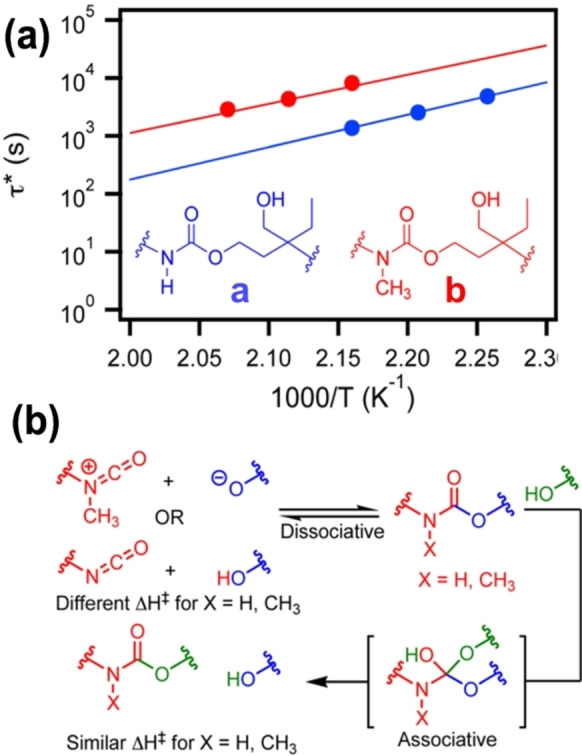
a) Arrhenius plots for the thermal activation of stress relaxation in **a** (blue) and **b** (red) are consistent with vitrimeric behavior. b) The similar activation energies for stress relaxation in **a** and **b** are suggestive of an associative transcarbamoylation mechanism (Adapted from ref. [80]).

The study discussed above introduced steric hindrance through the nucleophile (amine) of the reaction. However, steric effects can also be introduced through the electrophile (isocyanate) of the reaction. Fortman et al. reported a novel class of polyhydroxyurethanes derived from cyclic carbonates and amines without any catalyst.^[80]^ In their study, they introduced a steric effect through a methyl‐substituted carbamate which was unable to form neutral isocyanates (Figure 2b). This steric effect created a difference in pre‐exponential factors. Thus, they observed a slower stress relaxation in *N*‐methylurethane‐incorporated polymer which is bulkier compared to its non‐methylated polymer (Figure 2a). More importantly, they noted that the two distinct compounds (**a** and **b** in Figure 2a) undergo dynamic exchange through the same transcarbamoylation mechanism. The steric hindrance in *N*‐methylurethane has played a role in the nucleophilic addition to the methyl‐substituted carbamate by increasing the relaxation time. Nevertheless, more diversity of substituents is expected in future studies.

**Figure 3:**
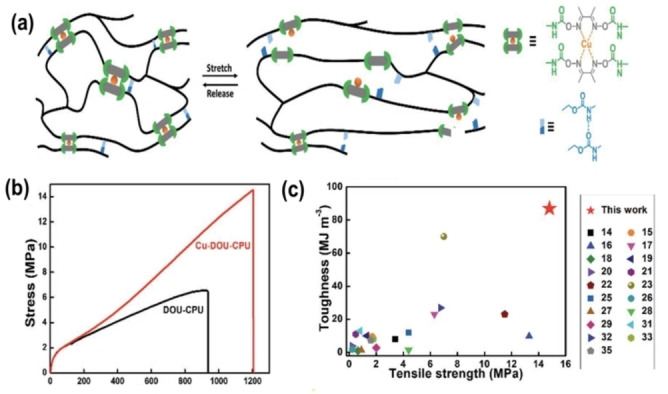
Mechanical properties of the Cu‐DOU‐CPU elastomer. a) Proposed mechanism of bond rupture and reformation in Cu‐DOU‐CPU during stretching. b) Tensile stress–strain curves of DOU‐CPU and Cu‐DOU‐CPU. c) Ashby plot of “toughness” and “tensile strength” of Cu‐DOU‐CPU and other room‐temperature self‐healing elastomers reported in the literature.[^57^, ^77^, ^87^, ^88^, ^89^, ^90^, ^91^, ^92^, ^93^, ^94^, ^95^, ^96^, ^97^, ^98^, ^99^, ^100^, ^101^, ^102^, ^103^] Cu‐DOU‐CPU exhibited the highest tensile strength and toughness (Adapted from the ref. [82]).

According to previous reports,[^31^, ^33^, ^69^, ^81^] the timescales of urea/urethane dynamic exchange can be fine‐tuned by controlling the steric hindrance in both nucleophile and electrophile. There are several other factors that can control the dynamic equilibrium of urea/urethane. Zhang et al. designed a self‐healing elastomer with outstanding mechanical properties.^[82]^ The elastomer consisted of triple dynamic bonds: reversible dimethylglyoxime–urethane, hydrogen bonding, and Cu coordination bonds (Figure 3a). Real‐time nuclear magnetic resonance and computational studies indicated that copper ions can significantly enhance the dynamic exchange of dimethylglyoxime–urethane bonds by acting as a catalyst. They reported that the Cu^2+^–imine coordination can recompense the penalty of hindrance created by methyl groups next to it. Due to the successive energy dissipation mechanism through reversible bonds, Cu^2+^‐incorporated materials demonstrated excellent tensile stress, toughness (Figure 3b, c), and self‐healing. Also, due to room temperature healing and excellent mechanical properties such as high tensile strength, good insulation, and high resistivity, this material could potentially be used as cable sheaths, hence demonstrating the tunability of material properties through synergistic effects.

Moreover, insertion of a vinyl group into urethane moieties has drawn attention as a way of controlling timescales of polymer materials (Scheme 3d). This insertion of a vinyl group enables urethanes to undergo associative transamination reactions at elevated temperatures without added catalyst.[^83^, ^84^] The effect caused by this insertion can stabilize the vinylogous urethane linkages, thermodynamically creating an α,β‐unsaturated carbonyl moiety. This moiety has high reactivity towards Michael‐type reactions with excellent hydrolytic stability.[^50^, ^85^] Exchange occurs though an associative mechanism as given in Scheme 3. For example, Denissen et al. designed a vinylogous urethane by introducing a double bond in‐between the nitrogen and the ester moiety of a urethane bond.^[86]^ The resulting materials could be fully reprocessed into a new material within minutes.

In 2017, the same team tuned the exchange kinetics of vinylogous urethane by using additives.^[104]^ The amine exchange of vinylogous urethane can be adjusted using Brønsted (or Lewis) acids or bases. The exchange rate accelerated in the presence of Brønsted or Lewis acid additives while base showed inhibitory effect. Fast stress relaxation (2 min) was demonstrated in the system with the acid catalyst compared to the uncatalyzed system (10 min) at 120 °C. Progress made in the area of dynamic polymers based on urethanes/urea bonds shows that dynamic character in resulting materials can be tuned by factors such as steric hinderance and electronic effect. The bulkiness of both the amine and isocyanates has a major impact on the dynamic properties of urethane polymers. When tuning the dynamicity of urethane polymers, *K*_eq_, *k*_−1_, and *k*_1_ parameters need to be well balanced to obtain high‐quality reversible dynamic polymers. Moreover, the effect of electron‐withdrawing and ‐donating groups as well as matrix effects on the adaptable timescale of dynamic urethanes/urea bonds remain underexplored.

## 4 Boronic Esters

**Scheme 4:**
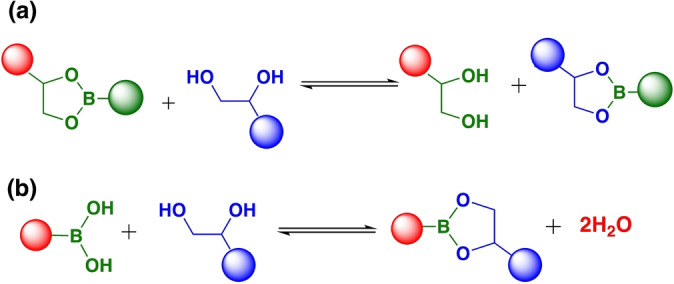
Reversible reaction of boronic esters from free diols and boronic esters: a) transesterification pathway and b) esterification via boronic acid.

Boronic esters have good thermodynamic stability with a B−O bond strength over 500 kJ mol^−1^, and excellent control over the kinetics of exchange.^[105]^ These boronic esters exchange reversibly through a transesterification mechanism between a boronic ester and free diols (Scheme 4a).[^105^, ^106^] Additionally, exchange can proceed through esterification between a diol and boronic acid.^[38]^ Boronic ester exchange is fast and sensitive to environmental conditions such as humidity, pH as well as the content of diols in the system.[^42^, ^107^, ^108^] Boron‐based polymers are potential candidates for catalysis, covalent organic frameworks,[^109^, ^110^] medicinal applications,^[40]^ sensors,^[111]^ electronic devices,[^112^, ^113^, ^114^] and have been used in organic synthesis.[^115^, ^116^, ^117^, ^118^, ^119^, ^120^]

**Figure 4:**
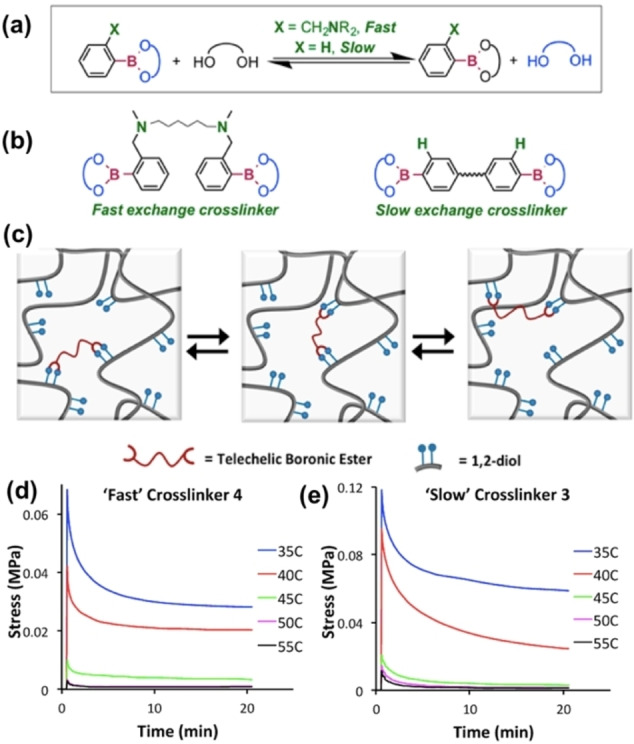
Design concept. a) Tuning a neighboring group to control the exchange kinetics of boronic ester. b) Design of diboronic ester cross‐linkers with tunable exchange kinetics. c) Dynamic exchange of boronic ester cross‐linkers affords dynamic materials. d,e) Stress relaxation data for 20 % diol PCO cross‐linked with diboronic esters at different temperatures (Adapted from the ref. [107]).

Transesterification of boronic esters can be tuned by sterics.^[121]^ In 2015, Cromwell et al. investigated the tunability of boronic ester bonds to control bulk dynamic properties (Figure 4c).^[107]^ Simply changing the neighboring groups (Figure 4a, b) caused small molecule exchange rate perturbations. Incorporating the *o*‐aminoethyl group accelerates the proton transfer between the leaving diol and the ammonium group with the nitrogen acting as a base during the transesterification.[^122^, ^123^] Two kinetically variable telechelic diboronic esters (Figure 4a) were designed by changing the neighboring groups. Even though both materials demonstrated malleability and reprocessability, these two kinetic variants exhibited differences in self‐healing due to the underlying bond exchange rates. Polymers containing the slowly exchanging unsubstituted linker showed significantly higher elastic modulus than the viscous modulus, confirming the inert nature of the linker. In contrast, the fast‐exchanging linker created a material that flowed, due to the rapid exchange kinetics. Additionally, stress relaxation studies confirmed the differences in bond exchange rates by showing a slow release of stress in a diphenylboronic ester cross‐linked system compared to the materials cross‐linked with di(*o*‐aminophenylboronic) ester (Figure 4d, e).

**Figure 5:**
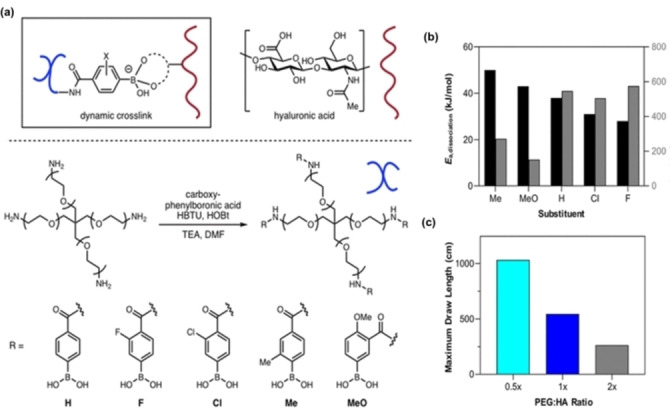
a) Proposed binding interaction between HA and branched PEG–PBA favored under basic conditions, and synthetic route for PEG–PBAs containing various chemical substituents on the phenyl ring (H, F, Cl, Me, MeO). b) Dependence of *E*_a_, dissociation, and maximum fiber draw length (of 10 experiments) on the PBA chemical substituent. c) Dependence of maximum fiber draw length (of 10 experiments) on the ratio of polymeric components, where *x* is defined as the molar ratio of PBA moieties (4 per PEG molecule) to HA disaccharide repeat units, ≈2 (Adapted from the ref. [124]).

Electronic effects also impacted exchange kinetics, where simply changing the substituent groups in boronic esters can be used to further tune the timescales of boronic ester exchange. To investigate this, Chu et al. reported structure–property relationships using withdrawing F and Cl groups as well as donating Me and MeO groups (Figure 5a).^[124]^ They observed reduced *E*_a,dissociation_ for electron‐withdrawing groups, indicating high dynamicity and the reverse was observed for electron‐donating groups. Unsubstituted or electron‐withdrawing groups (F and Cl) showed a strain at break higher than electron‐donating groups (Me and MeO) (Figure 5b). This high extensibility suggests that electron‐withdrawing groups improved the dynamic character of boron‐containing polymers due to the fast dissociation of bonds. Improved tensile stress and elastic modulus of an electron‐donating group (Me) might be due to slow dissociation of the dynamic bond which stiffens the polymer. Superior extensibility of these polymers is reported as ideal for biopolymer fibers. A study conducted by Yesilyurt et al. further confirmed that electron‐withdrawing groups accelerate the dynamic properties of boronic ester bonds by showing excellent self‐healing ability in fluorinated materials.^[125]^

In addition to the substituent effect, the molar ratio between polyethylene glycol polymers end‐functionalized with phenylboronic acids (PEG–PBA) and hyaluronic acid (HA) influenced the maximum draw length, tensile strength, and ultimate strain (Figure 5c). This method is a way to tune the dynamic properties of polymer materials without any synthetic effort. In 2018, Accardo and Kalow modulated photoresponsive viscoelastic hydrogels by controlling the stability of boronic ester.^[38]^ In their study, they observed the potential tunability of the equilibrium between diol and boronic ester by introducing F atoms adjacent to the boronic ester that is inspired by the previous studies conducted by Hecht et al.[^126^, ^127^] Their results indicated that F‐incorporated boronic ester stiffened faster and demonstrated high modulus compared to the non‐fluorinated boronic ester due to the enhanced binding affinity of the fluorinated boronic ester with diols. Furthermore, B−N coordination can shift the equilibrium toward the boronic ester product.[^107^, ^128^, ^129^] Therefore, it could be used to control the exchange rate of boronic esters. Song et al. reported the synergistic effect between boronic ester and B−N coordination.^[117]^ They observed an improvement of toughness and ultimate tensile strength by increasing the polymer nitrogen content. Nevertheless, reduced self‐healing efficiency was observed for high nitrogen containing materials due to decreased chain mobility. Despite the reduced self‐healing efficiency, damaged materials with high nitrogen content showed the highest tensile strength after healing.

Boronic esters are very sensitive to humidity/water content.[^40^, ^128^] In addition to molecular effects, water content can also be used to fine‐tune the timescales of boronic esters. For example, Song et al.^[117]^ reported enhanced self‐healing ability of boronic ester containing polyurethanes in the presence of water. This is due to the enhanced mobility of polymer chains through the partially dissociated B−N coordination, boronic esters, and hydrogen bonding. However, with increased moisture, the mechanical properties decreased due to the severe disruption of B−N coordination and hydrolysis of boronic ester bonds. In general, studies discussed above confirm that the reversibility of boronate ester bonds can be fine‐tuned by steric and electronic effects. Beyond structural modifications, water content can also influence the kinetics of the boronate ester exchange. In most of these studies, aryl boronic acids have been used as the model compound. Hence, the correlation between reversibility and the R group attached to the boron atom needs to be further studied in next‐generation polymer materials. Furthermore, in‐depth studies that will explore the coordination effect of the B atom are encouraged, especially going beyond B−N coordination.

## 5 Thiol–Michael Addition

**Scheme 5:**
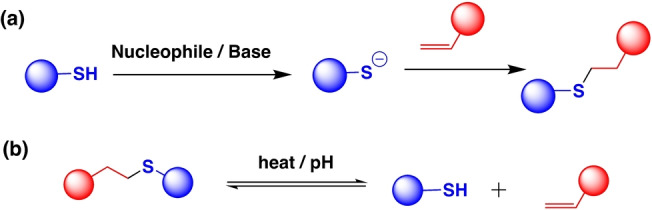
a) Base‐catalyzed or nucleophile‐catalyzed Thiol–Michael reaction. b) Reversible Thiol–Michael reaction under heat or high pH.

Thiol–Michael click chemistry, first reported in the 1960s,^[130]^ has been widely applied in polymer materials and is popular among organic chemists due to its efficiency.[^131^, ^132^] To the best of our knowledge, our group reported the first self‐healing and malleable materials using Thiol–Michael additions.^[133]^ The Thiol–Michael reaction involves 1,4‐addition of a thiol group into an electron‐deficient α,β‐unsaturated Michael acceptor (e.g., alkenes, alkyne) under base‐ or nucleophile‐catalyzed conditions (Scheme 5a).[^49^, ^132^] The reversibility of the Thiol–Michael reaction can be obtained by applying heat or at elevated pH (Scheme 5b).[^28^, ^134^]

Several small molecule studies have shown that Thiol–Michael addition and its dynamic reversibility can occur without catalyst at room temperature.[^131^, ^135^, ^136^] Herbert et al. have reported a catalyst‐free, room‐temperature benzalcyanoacetate‐based Thiol–Michael reaction in polymer networks.^[137]^ In their study, tunability was obtained by changing the electron‐donating or ‐withdrawing nature of Michael acceptors. They observed the highest strength for electron‐withdrawing nitro‐substituted Michael acceptors compared to electron‐neutral groups. Additionally, they found a new phenomenon, dynamic reaction induced phase separation, where the dynamic Thiol–Michael reaction led to distinct phases within the material. Besides electronic effects, our group showed that the polymerization method can also impact self‐healing and mechanical properties.^[138]^ Thiol–Michael crosslinked materials synthesized using reversible addition‐fragmentation chain transfer (RAFT) polymerization exhibited better self‐healing properties compared to materials synthesized via classical free‐radical polymerization. This was attributed to the uniform chains in RAFT, with minimal very short or very long chains in the system. This study suggests that not only structural changes, but also polymerization conditions and polymer structure can be used to tune the dynamic properties of the materials.

**Figure 6:**
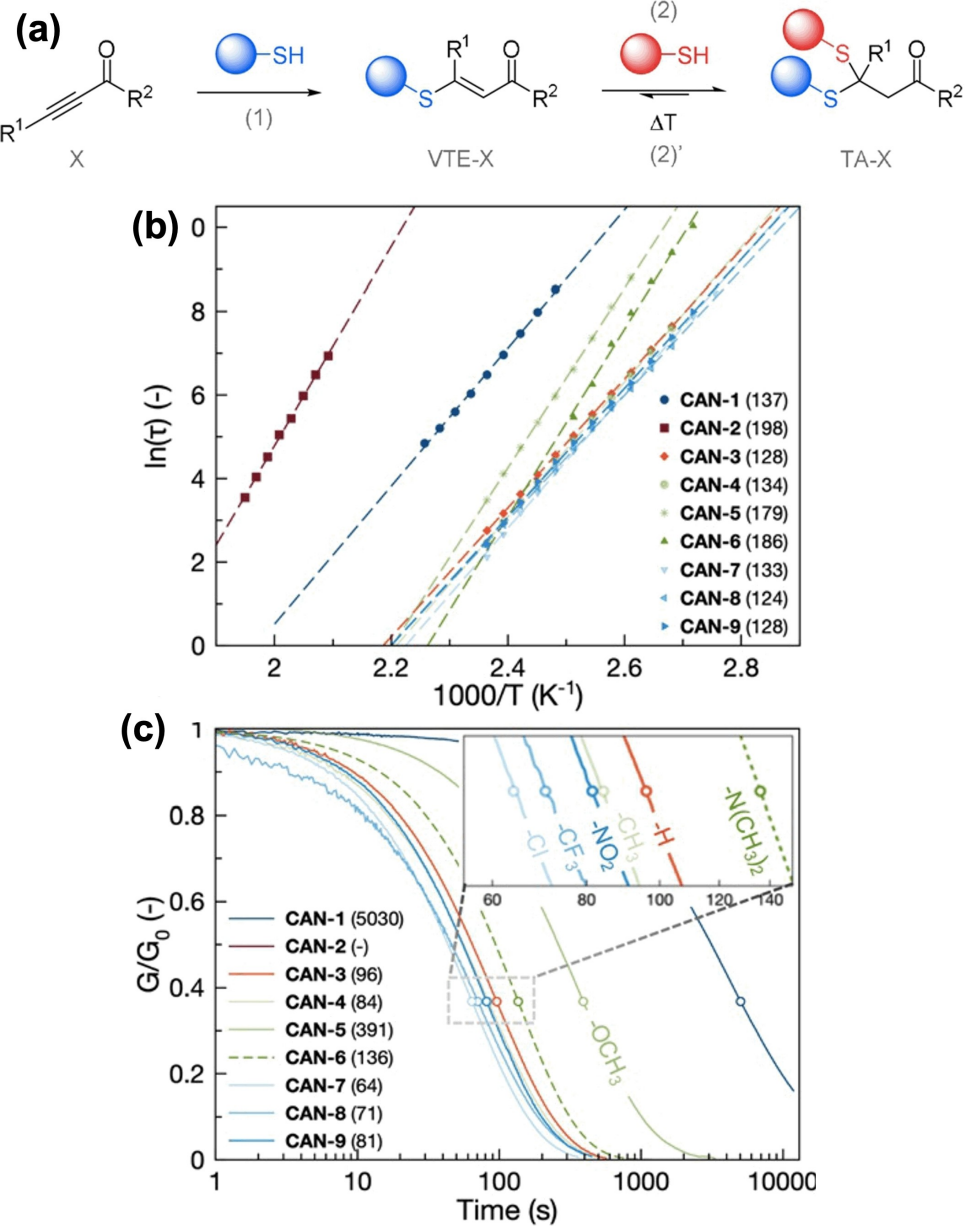
a) Synthesis of a thioacetal adduct (TA–X) via the base‐catalyzed double Thiol–Michael addition on alkynone precursors (X). (VTE–X) represents the β‐sulfido‐α,β‐unsaturated carbonyl intermediate. Reaction (2′) corresponds to the dynamic thiol exchange occurring at high temperature. b) Overview of Arrhenius plots with linear fits for all networks. Values in brackets represent the activation energy in kJ mol^−1^. c) Overview of stress relaxation curves at 130 °C for all networks. Values in brackets represent the relaxation time (at 1/e) in seconds at 130 °C. For CAN‐6 a Maxwell fit (dashed line) was created for clear comparison (Adapted from the ref. [139]).

According to Anslyn's model study, simple electronic effects can change the kinetics of Thiol–Michael reactions.^[135]^ With inspiration from this small molecule study, the tunability of Thiol–Michael reactions has drawn attention to tuning the properties of bulk polymers. For example, Van Herck et al. introduced covalent adaptable networks (CANs) cross‐linked via thiol–yne click chemistry (Figure 6a).^[139]^ They observed that *para*‐substituted electron‐withdrawing groups of a Michael acceptor enhanced the exchange rate while *para*‐substituted electron‐donating groups reduced the exchange rate, giving lower rate constants (Figure 6b). Accordingly, materials with electron‐withdrawing *para*‐substituted Michael acceptors relaxed faster (−Cl, −CF_3_, −NO_2_ and fastest for Cl substitution at 130 °C, 64 s) than the electron‐donating group incorporated materials (−OCH_3_, −N(CH_3_)_2_) (Figure 6c). The ketone functionality of alkynones and esters was replaced with sulfones to study the effect of the ketone replacement. Surprisingly, both model compounds acted as essentially irreversible static bonds. This showed that the choice of electronic substituent of the alkynone precursor can be used to modulate the exchange kinetics of the thiol–yne reactions. Moreover, bond reversibility also increased with higher temperatures, giving faster and full stress relaxation. Surprisingly, they also observed an unexpected faster relaxation time for *para*‐methylated phenylpropynone compared to the unsubstituted phenylpropynone. This was attributed to the enhanced mobility in the matrix which was corroborated by differential scanning calorimetry (DSC) results, highlighting the critical importance of considering temperature, matrix, and linkers when evaluating kinetics in materials based on Thiol–Michael chemistry.

Additionally, Long et al. recently investigated the effect of thiol substitution and functionality in Thiol–Michael reactions through monofunctional and multifunctional thiols.^[140]^ Their findings indicated that the rate of reaction depends on the thiol multiplicity. In systems with difunctional alkenes and difunctional thiols, secondary thiols showed faster reaction rates than primary thiols. In contrast, in tetrafunctional monomers (alkene and thiol), primary thiols showed faster reaction rates compared to secondary thiol. Therefore, thiol substitution is another way of controlling the rate of Thiol–Michael reactions and is a strategy that is useful in other dynamic chemistries. In summary, Thiol–Michael exchange can be manipulated by both structural and environmental effects such as substituent effect, matrix effect, and electron‐donating/withdrawing effects. Slower exchange and weaker association were observed for electron‐withdrawing groups attached to Michael acceptors at the vinyl group. In addition, functionality of the thiol and Michael acceptor has shown great impact on the reaction kinetics. Temperature can also simply be used to adjust the exchange kinetics of Thiol–Michael reactions without any synthetic effort.

## 6 Diels–Alder Reactions

**Scheme 6:**
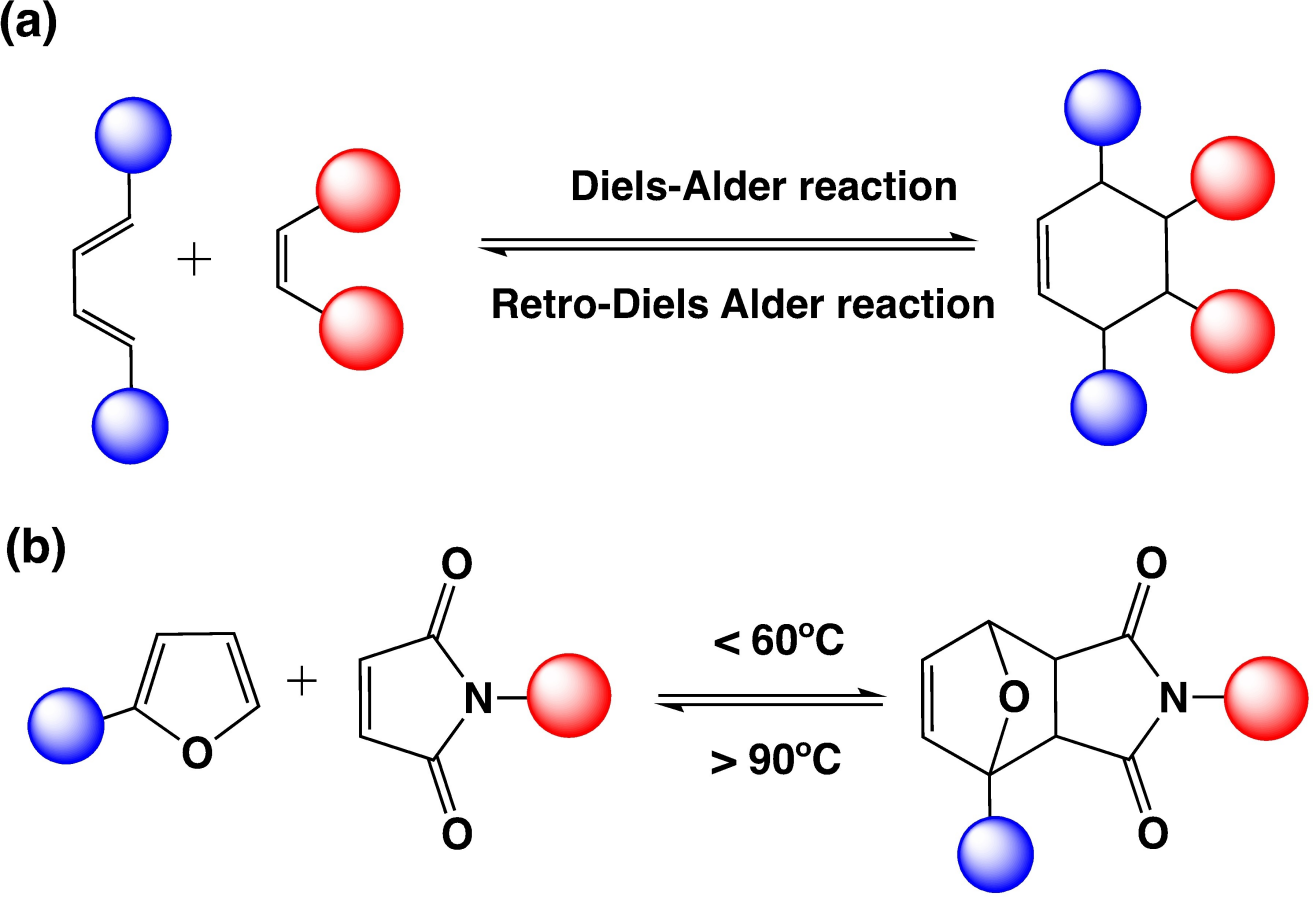
a) Thermoreversible Diels–Alder reaction. b) Retro‐Diels–Alder reaction between commonly used furan and maleimide groups.

The Diels–Alder click reaction involves a thermoreversible [4+2] cycloaddition reaction between a diene and dienophile which generates stereoselective cyclic compounds[^141^, ^142^] (Scheme 6a). The choice of the diene and dienophile can be modified for targeted applications in organic synthesis, coatings, adhesives, 3D printing, and drug delivery.[^143^, ^144^] Furan‐ and maleimide‐based compounds are the most common dienes and dienophiles, respectively, in materials applications (Scheme 6b).

This concerted reaction between furan and maleimide moieties forms the Diels–Alder adduct at or below 60 °C and the reverse reaction becomes significant above 90 °C.^[145, 146^ Diels—Alder adducts have been used in discrete polymers and bulk materials.^[147]^ Anthracene and maleimide showed reversibility at much higher temperature of 250 °C.^[148]^ Diels–Alder reactions can be controlled through electronic effects by changing the energy gap between the diene and the dienophile.^[149]^ This modulation of the reaction has been studied using small molecules by modifying the structure of the diene and the dienophile.^[148]^

In 2010, Canadell et al. investigated the stereoisomeric effect of retro‐Diels–Alder reaction.^[150]^ They investigated the influence of *endo*‐ and *exo*‐stereoisomers for the reversibility of the Diels–Alder reaction and the properties of the materials using DSC. They observed that retro‐Diels–Alder occurred between 20–40 K lower temperatures for the *endo*‐adduct compared to the *exo*‐adduct. However, further studies are needed to investigate how this affects the timescales and dynamic properties of Diels–Alder cross‐linked polymer materials.

Substituent effects are used to tune the reversibility and the dynamic properties of Diels–Alder adducts. Electron‐withdrawing dienophiles and electron‐donating dienes typically expedite the normal electron demand Diels–Alder reaction.^[143]^ In 2009, Reutenauer et al. reported Diels–Alder‐based room‐temperature self‐healing dynamic polymers. In their study, highly withdrawing cyano groups substituted molecules were employed as dienophiles. These cyano olefins facilitated the Diels–Alder reaction, resulting in room‐temperature reversibility. They observed excellent self‐healing at room temperature within ten seconds. In addition, Inglis et al. designed a reversible cross‐linked system through hetero Diels–Alder chemistry.^[151]^ They used an electron‐deficient dithioester as the dienophile and cyclopentadiene as the diene. They found that the retro‐Diels–Alder reaction took place around 5 min at >80 °C, indicating that electronic effects can be utilized as a tool for controlling the reversibility of the Diels–Alder reaction in dynamic polymers.

Furthermore, Xu et al. recently developed a reprocessable and self‐healable network with Diels–Alder adducts and Schiff base in one network.^[152]^ In their studies, Diels–Alder adducts dissociate at high temperatures reducing the cross‐link density of the polymer while lowering the hindrance of the networks to perform Schiff base exchange reactions. The associated Schiff base helps dissociated Diels–Alder bonds to anneal quickly. This combination gave materials that show excellent self‐healing properties. The Schiff base loading can be used to modulate the dynamic properties of the Diels–Alder cross‐linked network. Recently, Bailey et al. reported the effect of mechano‐lability of Diels–Alder adducts in material properties.^[153]^ They used a bulky cyclopropane attachment in a cyclopentadiene molecule that they believed affected the mechano‐lability of the resulting Diels–Alder adduct. Mechanoresistant cyclopentadiene–maleimide cross‐linked networks showed rapid gelation (<5 min) and 3‐fold increase in puncture resistance compared to the mechano‐labile traditional proximal furan–maleimide incorporated networks. Hence, this simple substitution provided a pathway to tune the dynamic properties of the Diels–Alder materials by going beyond traditional thermal tuning. In summary, the Diels–Alder reaction can be fine‐tuned by stereoisomeric effects of the retro‐Diels–Alder reaction, electronic effects, mechano‐lability of the Diels–Alder adduct, and synergistic effects. Electron‐withdrawing groups have shown more reversibility of the Diels–Alder reaction resulting in excellent self‐healing at room temperature. Hence, these parameters are useful for tuning the dynamic properties of bulk polymers such as self‐healing, stress relaxation, and creep recovery.

## 7 Transesterification

**Scheme 7:**
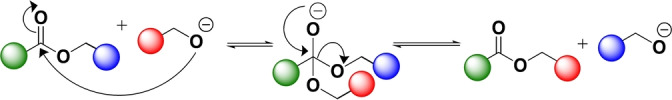
Reversible transesterification of ester linkages with free hydroxy groups under basic conditions.

Transesterification is a simple reaction in organic synthesis with many industrial applications.[^154^, ^155^] This involves the associative reaction of an ester and a free hydroxy group to form a new ester group (Scheme 7). Generally, transesterification requires catalysts such as Lewis acids, basic conditions, or elevated temperatures to improve the reaction speed.[^156^, ^157^] Many studies have been conducted to improve the mechanical properties of materials using transesterification reactions.[^158^, ^159^, ^160^]

In 2012, Capelot et al. reported a Zn‐catalyzed transesterification for the self‐healing and assembly of polymer networks.^[159]^ In their study they confirmed that the concentration of catalyst played a major role in controlling the kinetics of transesterification reaction. Materials with high catalyst concentration demonstrated higher strength compared to the lower concentration. In addition, their model molecule studies, and self‐healing test illustrated that the amount of hydroxy groups employed influenced the self‐healing kinetics. Materials with high ratio of epoxy:anhydride showed high force at break in their welding properties.

**Figure 7:**
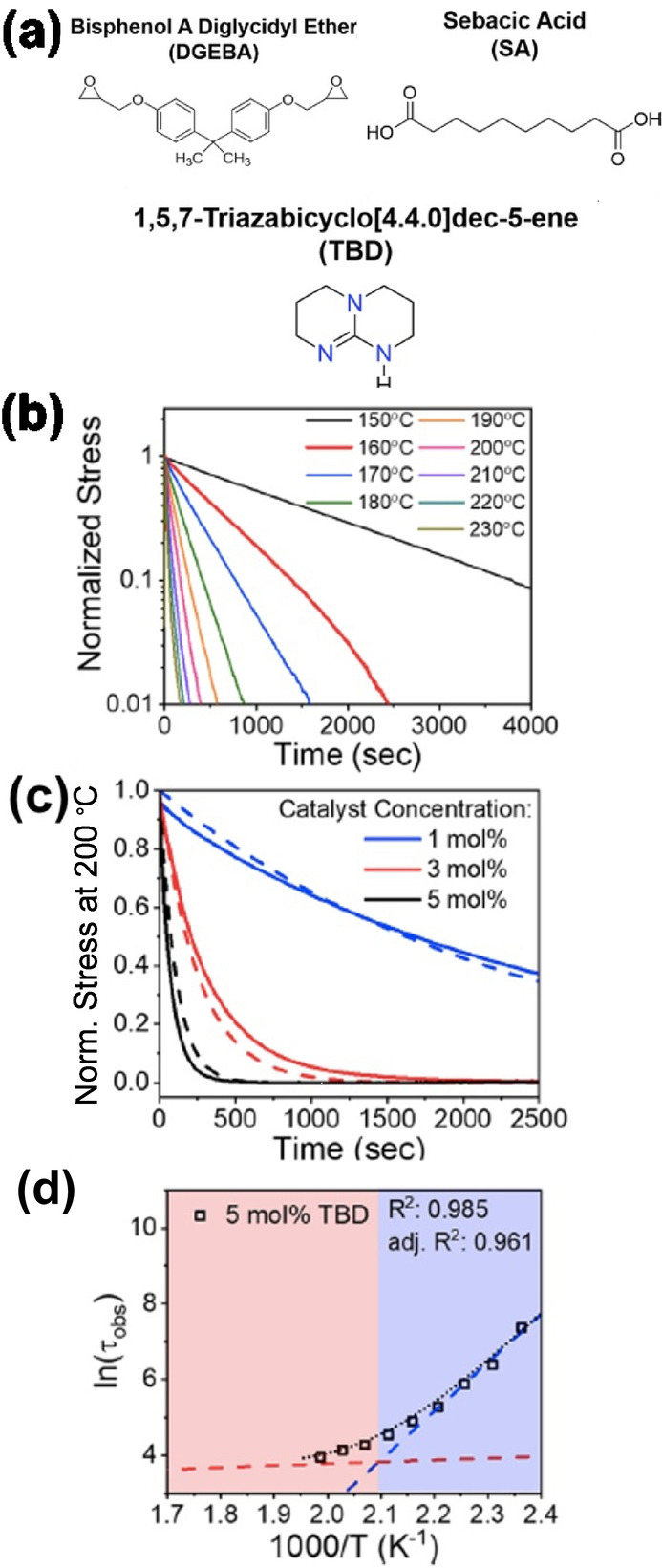
a) Chemical structures for all components within the vitrimer system are shown where TBD acts as the catalyst for transesterification at elevated temperatures. b) Stress relaxation results for a vitrimer with a 5 mol % catalyst concentration. c) Stress relaxation at a temperature of 200 °C shows that increasing catalyst concentration results in faster stress relaxation. d) Arrhenius plots are shown for 5 mol % catalyst concentration. The blue and red dashed lines indicate the two fits from the kinetic model for the chemically and diffusion‐limited regimes, respectively. The black dashed line indicates the kinetic model which exhibits good agreement with the experimental data, as confirmed by the *R* ^2^ and adjusted *R* ^2^ values listed (Adapted from ref. [161]).

In a separate study, Capelot et al. changed the concentration of Zn catalyst to fine‐tune dynamic properties of the bulk material.^[162]^ They observed the fastest stress relaxation at higher catalyst concentration. When the Zn catalyst was replaced with triphenylphosphine and triazobicyclodecene, triphenylphosphine showed full recovery in a creep recovery experiment than Zn(OAc)_2_ at 200 °C. Stress relaxation shows that Zn(OAc)_2_ and triazobicyclodecene are more efficient catalysts and they relaxed faster than triphenylphosphine at high temperature. In 2020, Hubbard et al. reported the effect of temperature and the catalyst amount on vitrimer transition temperature (*T*_v_) of transesterification reaction using diglycidyl ether of bisphenol A and sebacic acid with triazabicyclodecene (TBD) as the catalyst (Figure 7a).^[161]^ Their results agreed with the general trend that the stress relaxation time decreases with increasing catalyst or increasing temperature (Figure 7b–c). Interestingly, they identified a unique change in slope of the Arrhenius plot at higher temperatures which represents the *T*_v_ of the material (Figure 7d). At this temperature, the transesterification reaction is limited by diffusion within the material, and the relaxation time reaches a plateau.

**Figure 8:**
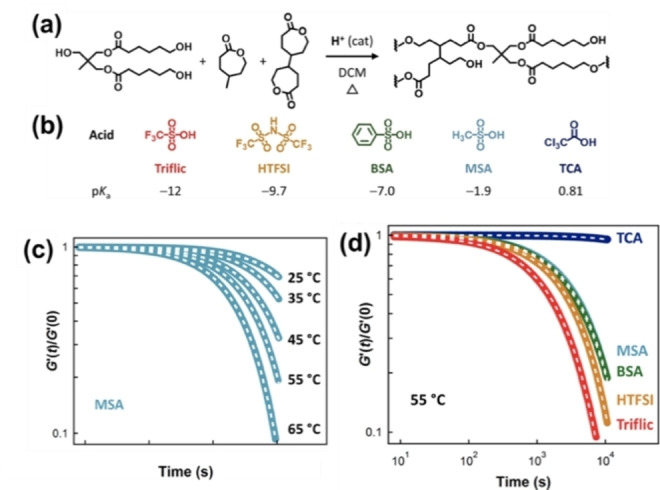
a) Schematic describing the ROP synthesis of low‐*T*_g_ polyester vitrimers. b) Brønsted acid catalysts used to promote ROP and subsequent exchange reactions via transesterification. p*K*_a_ values are referenced in H_2_O. c) Oscillatory rheology step‐strain–stress relaxation experiments on low‐*T*_g_ polyester vitrimer formulations containing Brønsted acid catalysts. Methanesulfonic acid measured at different temperatures. d) Step‐strain–stress relaxation experiments on low‐*T*_g_ polyester vitrimer formulations containing Brønsted acid catalysts with various acids compared at 55 °C (Adapted from ref. [158]).

Recently, Self et al. studied the effect of catalyst strength in esterification reactions using Brønsted acid catalysts (Figure 8a).^[158]^ Acids with different strength were used to tune the properties of materials (Figure 8b). Stress relaxation studies reveal that the strong protic acids improve network relaxation on the order of 10^4^–10^5^ s at 25 °C. They have shown that relaxation times depend on both temperature (Figure 8c) and acidity (Figure 8d). Overall, transesterification can be modulated by several factors: type of the catalyst, concentration of the catalyst, temperature, and the amount of hydroxy groups. Highly acidic catalysts (low p*K*_a_) and high temperature have shown improvement of the dynamic properties.

## 8 Imine Bonds

**Scheme 8:**
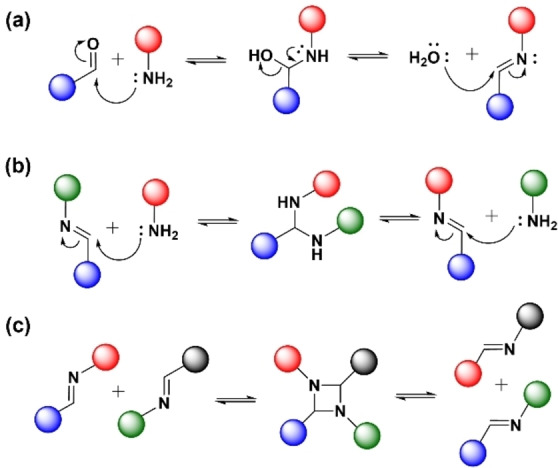
Reversible a) imine condensation and hydrolysis (dissociative exchange), b) transimination (associative amine–imine exchange), c) imine metathesis (associative imine–imine exchange).

Imines are formed by a condensation reaction between an aldehyde and a primary amine. This process is hydrolytically reversible through a dissociative mechanism (Scheme 8a).[^49^, ^163^] With or without a catalyst, imine exchange occurs in the absence of water via an associative exchange between a primary amine and an imine in a process called transimination (Scheme 8b).^[164]^ Additionally, through a catalyzed process, two imines can undergo an associative exchange to give two new imines via imine metathesis (Scheme 8c).^[165]^ Rapid imine exchange is considered a highly efficient reaction for the reversible formation and cleavage of covalent bonds, widely adopted in designing dynamic materials.[^166^, ^167^, ^168^, ^169^, ^170^, ^171^] The exchange dynamics of imine bonds can be tuned by heat, pH, water content, electronic effects, or polarity effects from the polymer matrix.[^72^, ^172^, ^173^]

**Figure 9:**
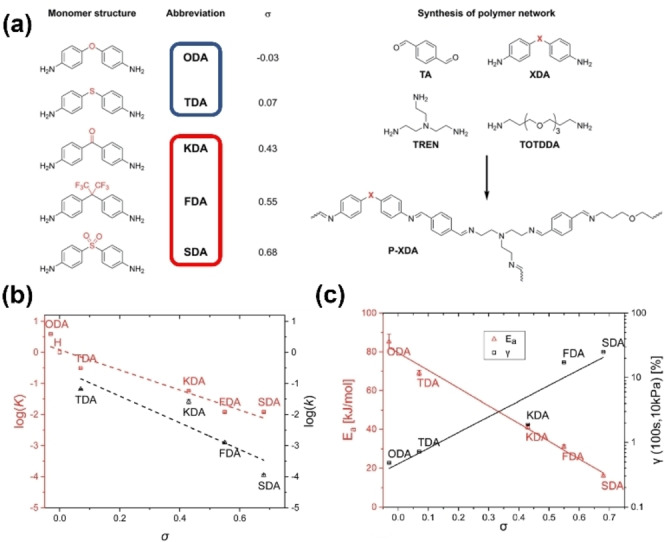
a) Tunable dianilines containing electron‐donating groups (blue box) and electron‐withdrawing groups (red box) (left) and synthesis of a typical polyimine network (right). b) Hammett plots of imine exchange reactions for studied tunable dianilines, showing the equilibrium constant (*K*, red squares) and the rate constant (*k*, black triangles) as a function of *σ*. c) Plot of *E*_a_ (red triangles) and *γ* (black squares) as a function of *σ* (Adapted from ref. [173]).

Schoustra et al. studied the electronic effect of dianiline monomers using the Hammett equation.^[173]^ They showed that the Hammett parameter (*σ*) of tunable dianiline monomers (XDAs) controlled the relaxation time, kinetic activation energy (*E*_a_), topology freezing (*T*_v_), material creep, and glass transition temperature (*T*_g_) of polyimine networks, consequently allowing for easy tunability of dynamic mechanical and thermal properties (Figure 9a). Kinetic data obtained from Hammett plots (Figure 9b) revealed that increasing *σ* led to significantly decreased reaction rates of imine exchange reaction in small molecule studies. Figure 9c gives the trends observed in polymer materials containing XDA substituents. Increasing the electron‐donating effect increased *E*_a_ and increasing the electron‐withdrawing effect decreased *E*_a_. This work confirms that donating groups stabilize imine bonds and consequently increase the energy required for the exchange reaction. Similarly, creep tests revealed that increasing *σ* led to an increased amount of strain (*γ*) (Figure 9c, black squares). This implies that regardless of the pathway (associative or dissociative) for imine bond exchange reactions, Hammett‐equation‐based principles can be used to quantitively correlate molecule‐based physical parameters to macroscopic properties of materials. In a separate study, the same research group reported that the rate of imine exchange reactions can be significantly enhanced when polar functionalities are placed near reactive imines in the matrix.^[72]^

Stefano et al. also showed that sterically unhindered imines undergo faster exchange reactions with primary amines compared to sterically hindered amines under very mild reaction conditions.^[165]^ Polyimine cross‐linked thin films were also reported to behave like thermosets at room temperature which upon application of moisture or heat will result in polymer networks that could be easily reprocessed like a malleable thermoplastic and also efficiently undergo macroscopic stress relaxation.^[174]^ Chao et al. reported the tuning of properties in polymer networks composed of imine cross‐linkers either in gel form or as bulk materials. Solvent choice modulated imine bond exchange rates and thus the dynamic properties of resulting polymer gel networks. This study provided insights on the molecular and kinetic basis for the macroscopically observed dynamic properties in imine‐linked polymeric networks.^[170]^ Summarily, the timescale of exchange in imine bonds can be tuned by systematic variation of reaction dynamics through a suitable selection of monomers that make up aromatic imine building blocks and by utilization of polar groups near reactive imine species. Materials based on polyimine offer a pathway for making tunable and recyclable materials suitable for a broad range of applications such as fire retardants,^[175]^ 3D printing,^[176]^ coatings,^[177]^ and electronic skins.^[178]^

## 9 Ionic and Coordination Bonds

Among the conceptually simplest noncovalent interactions are electrostatic interactions between cations and anions. In a recent study, Gong et al. tuned the dynamic exchange rate of hydrogels containing both permanent covalent and exchangeable ionic cross‐links.^[179]^ Modifying the free salt concentration in the network modulated the dynamics of the ionic cross‐links, with higher salt concentrations weakening the ionic bonds through electrostatic screening. Effective dynamic bonds that exchange on the timescales of the experiment or mechanical challenge improved fracture and fatigue properties of the material. This enhancement in toughness and fatigue arises from the DNCBs dissipating energy under load.

Metal–ligand coordination can be significantly more specific and tunable than purely electrostatic interactions. The development of metallosupramolecular polymers (MSPs) was first reported by Rowan et al.[^98^, ^180^, ^181^] MSPs have unique properties including luminescence, dielectrics, magnetism, electronics, optics, stimuli‐responsiveness, catalysis, and shape memory.[^48^, ^68^] The strength of coordination bonds is tunable within the range of 25–95 % of a covalent C−C bond.^[182]^ Careful selection of metal–ligand combinations can be used to achieve dynamic coordination bonds suitable for autonomous,^[87]^ optically,[^180^, ^183^] solvent‐,^[184]^ and thermally^[185]^ induced self‐healing. In designing MSPs, the choice of a dynamic complex is essential in obtaining a high degree of polymerization. The choice of ligands and metals to obtain different binding constants has been discussed extensively in excellent reviews from Zhang et al.^[48]^ and Würthner et al.^[186]^

**Figure 10:**
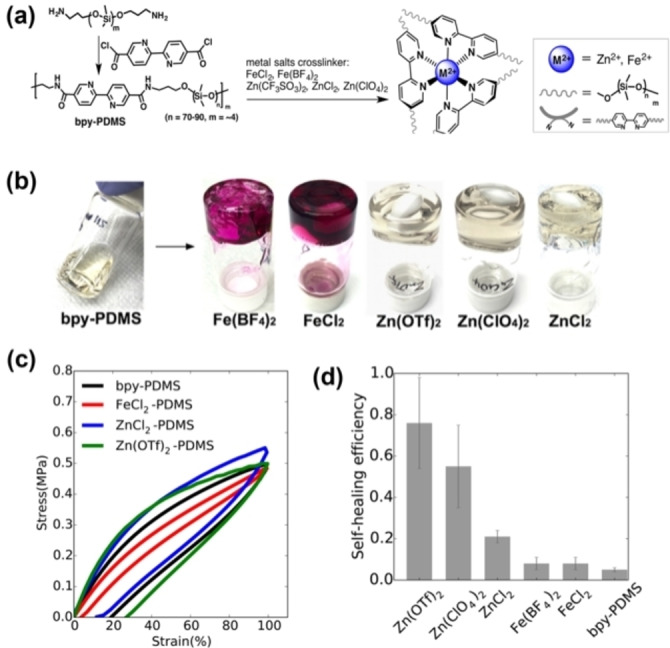
a) The synthetic route for metal salts cross‐linked PDMS. b) Photos of gelation of bpy–PDMS toluene solution (70 mg mL^−1^, 3 mL) upon addition of various metal salts in methanol as cross‐linkers (0.18 M/L, 50 μL). c) Stress–strain curves of polymers bpy–PDMS, FeCl_2_–PDMS, ZnCl_2_ and Zn(OTf)_2_–PDMS, with a displacement rate of 5 mm/min. d) Bar graph summarizing self‐healing efficiencies of all polymers at ambient condition after 48 h (Adapted from ref. [98]).

Bao et al. demonstrated an application of MSPs as self‐healable dielectric materials.^[98]^ In their study, they incorporated bipyridine motifs into linear polydimethylsiloxane (PDMS) chains subsequently cross‐linked via metal–ligand coordination between bipyridine moieties and different transition‐metal ions of Zn^2+^ and Fe^2+^ (Figure 10a,b). Cyclic stress–strain experiments show that functionalized PDMS exhibited different extents of hysteresis during loading and unloading (Figure 10c). Zn^2+^‐based cross‐linkers gave much larger hysteresis which was consistent with stress relaxation experiments wherein faster stress relaxation was observed in Zn^2+^‐cross‐linked materials compared to Fe^2+^ materials. Zn^2+^ had superior energy dissipation when placed under mechanical stress due to two factors. Firstly, the Zn^2+^−N(bipyridyl) bond is kinetically more labile than the Fe^2+^−N(bipyridyl) bond; and secondly, Zn^2+^ has the natural ability to assume both tetrahedral and octahedral geometries, possessing another path to minimize stress. Hence, the choice of metal ions with different coordination characteristics is a useful tool for tuning polymer dynamics to obtain different self‐healing efficiencies, with Zn(OTf)_2_ showing ≈80 % healing efficiency (Figure 10d).

**Figure 11:**
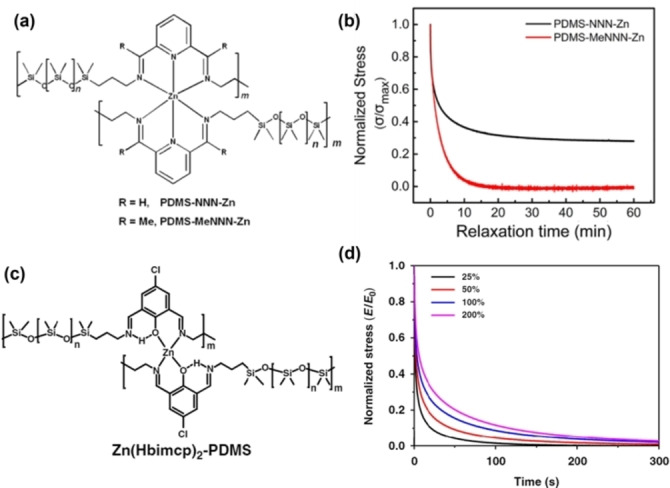
a) Structure and properties of polymer films. b) Stress relaxation curves of films primarily stretched to 100 % strain and then allowed to relax for 60 min at room temperature (Adapted from ref. [187]). c) Possible structure for [Zn(Hbimcp)_2_]^2+^ complex. d) Normalized stress relaxation curves under different strain. (Adapted from ref. [188]).

In 2018, Li et al. reported the electronic effect of side groups and the impact of subtle modifications in coordination complexes of linear PDMS polymers, cross‐linked with similar but distinct Zn^2+^–diiminopyridine complexes (Figure 11a).^[187]^ They showed that the presence of methyl side groups led to a small decrease in strength but significant improvement in elasticity and toughness of PDMS‐MeNNN‐Zn. Faster ligand exchange processes were supported by normalized stress relaxation curves which showed that PDMS‐MeNNN‐Zn released stress much faster than PDMS‐NNN‐Zn (Figure 11b). This suggests that side methyl groups can induce substantial perturbations to coordination bonds, weakening the interaction and increasing kinetic lability which favors self‐healing and faster relaxation of stress.^[98]^ Similarly, Bao et al. presented the cross‐linking of a linear PDMS polymer via Zn^2+^ coordination (Figure 11c).^[188]^ In their study, they found that longer healing time led to higher recovered fracture strain and ≈99 % healing efficiency at room temperature, strongly indicating dynamic exchange of metal–ligand coordination bonds. This was further observed in substantial relaxation of stress (Figure 11d). Therefore, the adaptable timescale in coordination bonds can be tuned by the choice of metal cations (sometimes with different oxidation states) and counteranions to control bond strength.

In 2005, Yount et al. reported metal–ligand coordination between bis‐Pd^II^ and Pt^II^ organometallic cross‐linkers and poly(4‐vinylpyridine) in DMSO.^[189]^ In their model study, they observed higher dissociation rate constants for the Pd system than for the Pt system, demonstrating a higher modulus at lower frequencies compared to the Pd system. For polymers with the larger Et group, the plateau in the shear storage modulus extended to lower frequencies than with the cross‐linkers based on the smaller Me group. In addition, the nucleophilicity of solvent (DMSO, DMF, or DCM) towards the metal was found to control the ligand exchange rate in the networks, due to the dissociative pathway.

A literature report from Guan et al.^[190]^ showed that it is possible to tune the kinetics of coordination bonds for simultaneous control over both polymer cross‐link structure and responsiveness of the resulting bulk material by varying the dynamic association of monodentate and weak ligands with different transition metals. Additionally, subtle modification of coordination complexes to influence thermodynamic stability and kinetic lability of cross‐linking sites significantly contributes to the mechanical and self‐healing properties of the resulting polymers, which can be useful in designing energy absorbing materials for armored clothing, sportswear, etc.

## 10 Hydrogen Bonding

Hydrogen bonds (H‐bonds) are one of the most widely employed DNBs. H‐bond cross‐linked polymers have been used as electronic skins,^[191]^ binders for battery anodes,^[192]^ strain sensors,^[193]^ stretchable electrodes,^[194]^ intelligent devices,^[55]^ and gas‐separation membranes.^[98]^ A reversible H‐bond cross‐linked polymer based on 2‐ureido‐4‐pyrimidone (UPy) units was first reported by Meijer et al. in 1997. UPy was used to cross‐link a trifunctional copolymer of hydroxy‐terminated poly(propylene oxide) by forming quadruple H‐bonds^[195]^ with improved strength and stretchability. Since then, incorporation of H‐bonds as cross‐linking motifs in polymeric materials has become popular with several examples including urea functionalization of polydimethylsiloxane,^[100]^ pyrimidine‐derived multi‐amine molecules as H‐bond cross‐linking agents for poly(vinyl alcohol),^[196]^ thiourea units in poly(ether thiourea),^[197]^ a H‐bonding complex between 2,4‐diaminotriazine and cyanuric acid,^[198]^ supramolecular H‐bonding in polyamide based‐elastomers,^[199]^ amide groups as side chains in polyesters,^[200]^ or part of the backbone in poly(ester amides),^[201]^ as well as hydrazides in poly(ester hydrazides)^[202]^ and poly(ester dihydrazides).^[203]^

**Scheme 9:**
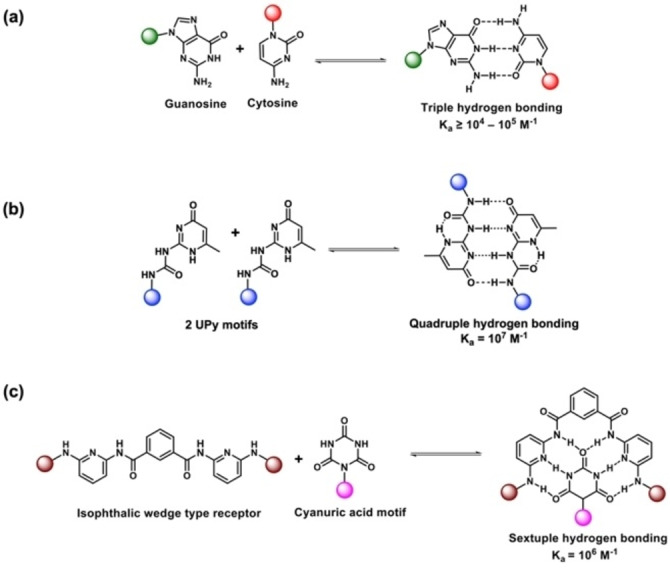
Examples of hydrogen bonding arrays and their binding constants: a) Triple hydrogen bonding via guanosine–cytosine in CDCl3; b) Quadruple hydrogen bonding via UPy–UPy in CDCl_3_ (Inspired by ref. [210]); (c) Sextuple hydrogen bonding via cyanuric acid–isophthalic receptor derivatives (Inspired by ref. [211]).

UPy motifs are the most commonly employed due to their ease of synthesis, readily available derivatives for polymer functionalization, and high dimerization constant of 10^7^ M^−1^ in solution^[204]^ (Scheme 9). The high dimerization constant corresponds to ≥40 kJ mol^−1^ required to completely dissociate a UPy dimer. Although lower than a typical covalent bond, the free energy sufficient for breaking a UPy dimer is similar to the energy required for protein unfolding.^[205]^ Therefore, cross‐linking polymeric materials via UPy dimerization results in improved mechanical strength, toughness, and high extensibility owing to the continuous dissociation or exchange of UPy dimers when the resulting materials are stretched.

H‐bond cross‐linked polymer networks can be categorized as: (i) self‐association or self‐assembly and (ii) externally added cross‐linkers. For self‐associative H‐bonded polymer networks, materials are synthesized by chemically incorporating H‐bonding motifs to polymer main chains,^[206]^ side chains,^[207]^ telechelic chain ends,^[208]^ or via self‐complementary multiple interchain H‐bonding.^[209]^ The properties and timescales of H‐bond cross‐linked polymers can be tuned by varying the underlying microstructures of polymer backbones (for both steric and electronic effects), exploring different chemical components of cross‐linking motifs, varying the degree of cross‐linking, and by changing experimental conditions.

**Figure 12:**
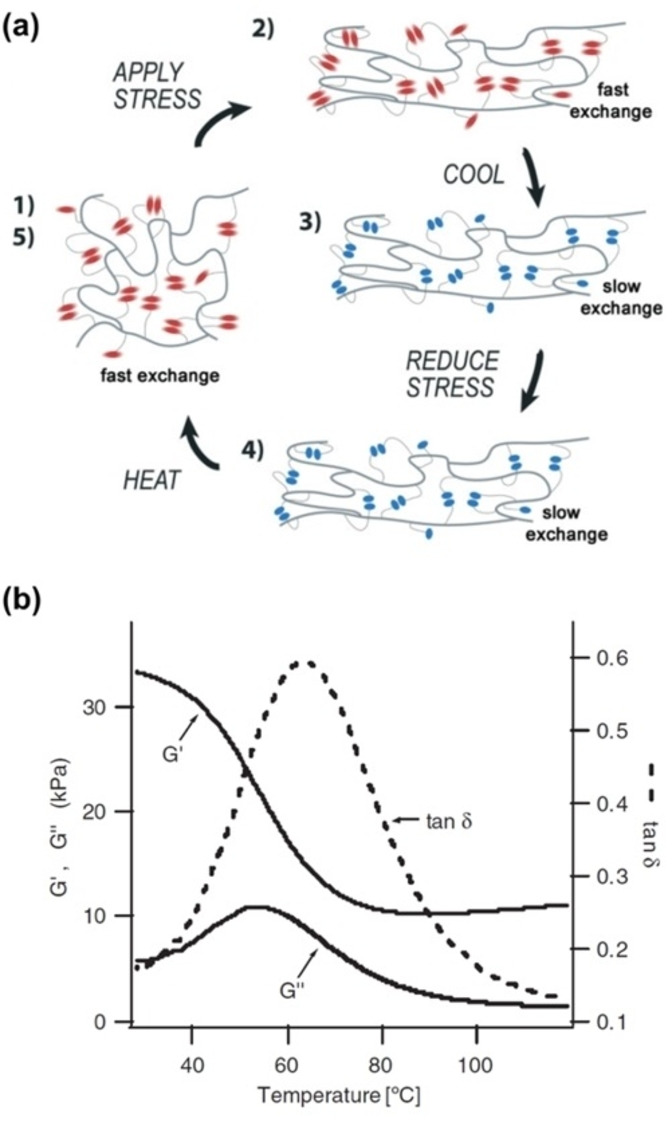
a) Cartoon of a proposed shape‐memory mechanism involving thermoreversible H‐bonding. Colored side‐groups represent H‐bonding groups in the hot (red) and cold (blue) states, and the darker lines represent the lightly cross‐linked covalent network. b) DMTA scan (2 °C min^−1^) at constant frequency (0.1 Hz) of a sample containing 2 mol % of UPy side‐groups (Adapted from ref. [212]).

In 2007, Anthamatten et al. reported that the dissociation dynamics of UPy quadrupole H‐bonds in poly(butyl acrylate) cross‐linked by trifunctional trimethacrylate can be used to tune the mechanical relaxation of polymer networks.^[212]^ They showed that H‐bonding interactions alone can be used to stabilize mechanically strained states in elastomeric materials (Figure 12a). Upon heating, the material strain is completely recovered and control samples without UPy motifs did not show these properties. Therefore, at relatively low temperatures, H‐bonds contribute to the storage modulus and take a long time to exhibit self‐healing properties.^[213]^ However, at high temperatures (>40 °C), the lifetimes are quite short, and they are unable to contribute to mechanical damping or material elasticity. Dynamic mechanical thermal analysis (DMTA) data confirmed the shape memory studies and give a clearer picture of H‐bonding in an elastic network (Figure 12b).

**Figure 13:**
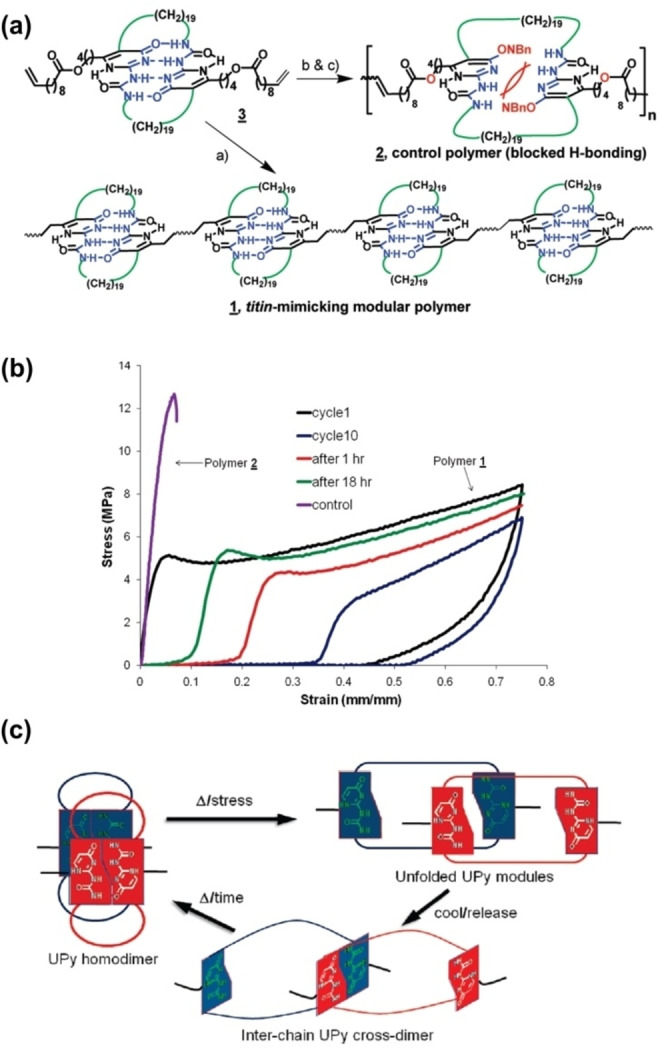
a) Synthesis of biomimetic linear modular polymer **1** and control polymer **2**. b) Stress–strain curves for polymer **1** and control polymer **2**. c) Proposed molecular mechanism accounting for the rare combination of mechanical properties observed in polymer **1** (Adapted from ref. [213]).

The effect of steric interactions and the polymer matrix on H‐bond interactions was reported by Guan et al. using an amorphous polymer that contains sterically hindered H‐bond cross‐links.^[213]^ Polymer **1** was designed to ensure the polymer backbone contains no H‐bonding or interaction sites, while the control material contained *ortho*‐nitrobenzyl (NBn) which was added to sterically hinder H‐bonding, hence the resulting polymer **2** lacks the ability to form UPy dimers (Figure 13a). Without reversible H‐bonding capability, polymer **2** was observed to differ drastically from the polymer **1** by showing poor adaptive properties, extensibility, and toughness (Figure 13b). This confirms the effect of steric interaction in hindering easy access of H‐bonding units. The presence of bulky and rigid UPy modules within flexible alkene spacers as shown in polymers **1** and **2** leads to polymer chains that are easily trapped in entanglements and are responsible for properties such as temporary fixation of polymer shape as shown in Figure 13c. Interlocking of polymer chains via molecular steric interactions has been reported by Tsui et al. to enhance material ductility while simultaneously increasing strength and stiffness.^[214]^ Therefore, the polymer matrix plays a role in tuning the dynamic nature of H‐bonding cross‐linked materials. Additionally, our group reported that the increased association constant of UPy units in non‐polar poly(ethyl acrylate) matrixes (giving higher‐quality H‐bonds) is more important for mechanically tough materials than the total H‐bonding in poly(hydroxylethyl acrylate) matrixes (giving a greater quantity of H‐bonds).^[204]^ Hence, dynamic H‐bond strength of association can be employed in tuning the mechanical properties of materials.

**Figure 14:**
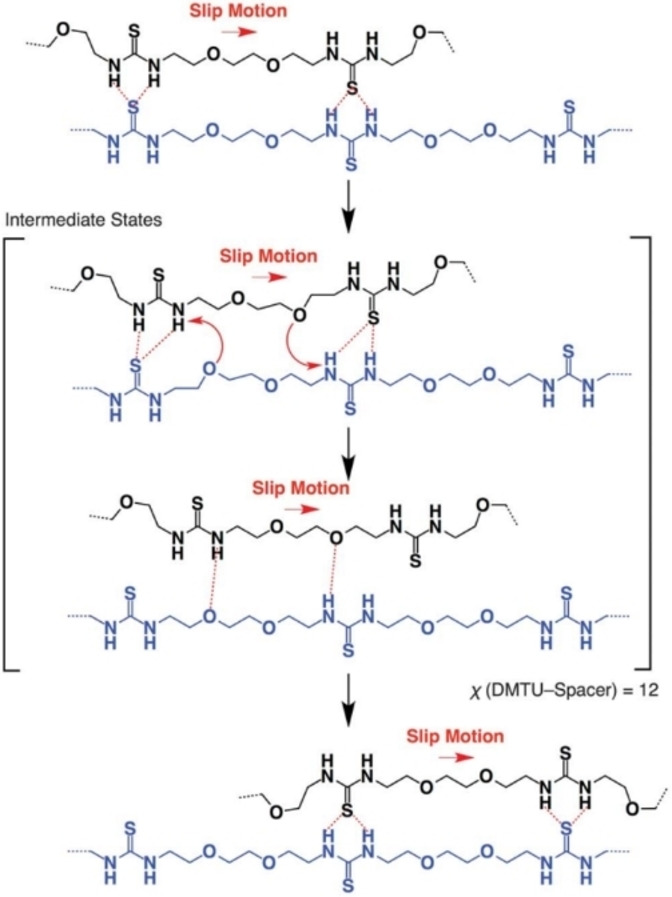
Proposed mechanism of how the exchange of H‐bonded thiourea pairs is enhanced (Adapted from ref. [198]).

According to Yanagisawa et al., the exchange of H‐bonding pairs can also be enhanced by polymer chain slipping and interpenetration of rapidly exchanging H‐bonded pairs. (Figure 14). Overall, the timescale of dynamic H‐bonding and self‐healing can be tuned by using experimental temperature to modulate the cross‐linking lifetime of materials. Also, utilizing steric influence through polymer microstructure strength of association, and multiplicity of high‐quality dynamic H‐bonds are valuable ways to control the timescale of H‐bonding in cross‐linked polymer materials. Additionally, compared to DCBs, DNBs (e.g., H‐bonding and coordination bonds) often result in materials with higher kinetic mobility of dynamic cross‐links and as such can easily give extremely low *T*_g_ and an impressive low‐temperature self‐healing.

## 11 Advantages and Disadvantages of Different Dynamic Bonds

Each bond has its unique features and drawbacks. For example, urethanes are cost‐effective and can be synthesized from readily available precursors. However, the toxicity of isocyanates is problematic. This can be minimized using non‐toxic precursor compounds such as cyclic carbonates instead of isocyanates. Boronic esters are more biocompatible but are greatly affected by humidity and acids; hence, they can undergo cross‐coupling and are difficult to handle. Thiol–Michael exchange is a high‐yield reaction with high selectivity. However, some of the precursors are toxic and thiols have unpleasant odors which can be highly inconvenient during handling. The Diels–Alder reaction is commonly used in biological applications as it shows water compatibility, high stereoselectivity and regioselectivity without any catalyst. A challenge associated to Diels–Alder exchange is the possibility of cross reactions with other nucleophiles such as amino groups. Transesterification is a cost‐effective and well‐controlled reaction which gives robust and processable polymers. However, most transesterification reactions require high catalyst loading and high processing temperatures. Dynamic imine bonds are often studied because they are readily obtained from condensation between an aldehyde and an amine under ambient conditions, giving off only water as by‐product and without the need for a catalyst. However, reversibility in imine bonds can be prevented when the imine bond formed is highly stable and also by removing water from the system.^[173]^ For dynamic noncovalent interactions, coordination bonds often result in self‐healing materials that are not significantly affected by surface ageing compared to self‐healing materials based on hydrogen bonding.^[57]^ Additionally, materials based on metallosupramolecular interactions are kinetically labile and thermodynamically stable hence facilitating effective self‐healing even at very low temperatures.^[178]^ One other advantage of M−L interactions is that they can be used to engineer ligand‐containing polymers without the need for synthetic modification of the polymer backbone. Many monomers contain hydrogen bonds facilitating incorporation of some hydrogen bonds, however, they are typically weaker than other dynamic interactions, necessitating multiple coupled hydrogen bonds, which can be synthetically challenging.

## 12 Conclusions and Outlook

This Review focused on established and emerging strategies for fine‐tuning the mechanical properties of materials. Both dynamic covalent and dynamic noncovalent bonds marked notable advances in commodity polymers. Among the urea/urethanes, Thiol–Michael exchange, Diels–Alder reactions, boronic esters, hydrogen bonds, coordination bonds, imine bonds, and transesterification reactions have attracted significant attention from polymer chemists. Most of these dynamic bonds require a catalyst or various environmental conditions to enhance or diminish the reversibility of the bond. Tuning the reversibility of dynamic bonds is a way of tailoring the mechanical properties which can adapt materials towards applications with unique properties. Electronic effects, steric effects, matrix effects, catalyst loadings, and solvent effects are the most common pathways to control the dynamic characters of small molecular kinetics and as such serve as an inspirational guide to tune dynamic properties of polymers. Although the choice of dynamic interaction can be used to influence the properties of polymer materials, a better and more fine‐tuned approach for designing dynamic materials from the bottom up with specific target properties is still underexplored.

Introduction of electronic perturbations through the electron‐withdrawing/donating groups is a simple way of tuning the kinetics of bond reversion. Depending on the position where the electron‐withdrawing/donating moiety is attached, the rate of reversibility will be altered. For example, rapid exchanges can be obtained from electron‐withdrawing groups (F, Cl) attached near the carbonyl of Michael acceptors while the opposite trend is observed for electron‐withdrawing groups attached near the vinyl group. Stress relaxation, self‐healing, creep recovery, and the strength of the materials can be modulated using these electronic effects. In addition, steric effects play a major role in fine‐tuning properties of materials. Sterics can weaken the bonds and reduce the conjugation effects of dynamic moieties such as urea/urethane bonds. The size of the bulky group should be optimized to obtain sufficient *k*_−1_ and *K*_eq_. The solvent effect and matrix effect have been less studied in this field and need to be further explored. These types of strategies including the temperature effect can be applied to regulate the reversibility without any synthetic efforts. Besides, the remarkable influence from the combined interactions between different moieties has been less studied and needs to be further expanded.

Dynamic polymers have potential applications in medicine, electronics, agriculture, paints, and transportation. Slow reversible dynamic chemistries are potential systems for material applications such as tires, coatings, and adhesives that need a more static nature but also need some reversibility for repairing cracks, scratches, or damages. Slow exchange chemistries ensure the long‐term durability of materials without trading off mechanical properties. In contrast, fast exchanging chemistries are beneficial for applications that need a transient nature such as drug delivery in medicinal chemistry, to release fertilizers in agricultural fields and materials that need biodegradability or recyclability. In addition to the magnitude of the reversibility, catalyst‐free room‐temperature healable materials are ideal for many industries that require stimuli‐responsiveness but no harsh conditions and toxic chemicals.

Modulation of the kinetics in dynamic bonds will provide new insights to produce sustainable recyclable materials using mild conditions. New principles need to be developed for fine‐tuning the kinetics of dynamic reversibility in bulk systems. In addition, computational studies will be more important for the development of tunability in dynamic materials by predicting their dynamic behavior before laboratory experiments. Although this Review provided guidelines for tuning dynamic bonds, the quantitative prediction of materials from identified small molecule systems is less well understood. Further research in that area will ultimately allow for control over the macroscopic properties of dynamic materials by channeling changes at the molecular level. Overall, this review highlights recent developments in tuning dynamic bonds (in polymeric systems) and hence serves as a guide for the design of sophisticated dynamic materials via simple structural and environmental modifications.

## Conflict of interest

The authors declare no conflict of interest.

## Biographical Information

*Shiwanka V. Wanasinghe earned her B.Sc. in Chemistry at University of Kelaniya, Sri Lanka in 2017. She is currently a Ph.D. candidate at the Department of Chemistry and Biochemistry, Miami University. In her research, she focuses on developing single networks and interpenetrated networks with dynamic bonds and works with RAFT and ATRP systems in the group of Prof. Dominik Konkolewicz*.

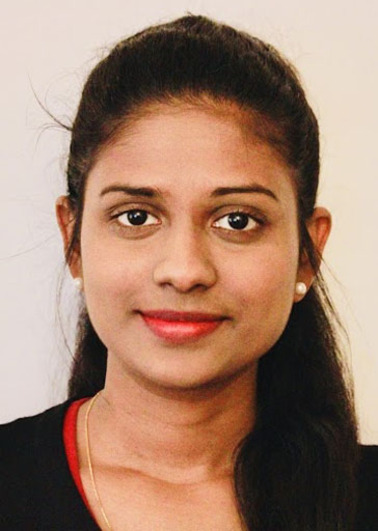

## Biographical Information

*Obed J. Dodo received his B.Sc. degree in Chemistry from Kaduna State University in 2017. Currently, he is a fifth year Ph.D. candidate working with Prof. Dominik Konkolewicz at Miami University. His research interest focuses on utilizing macromolecular engineering to design and synthesize bulk dynamic polymer materials covalently enhanced with carbon nanotubes for targeted applications*.

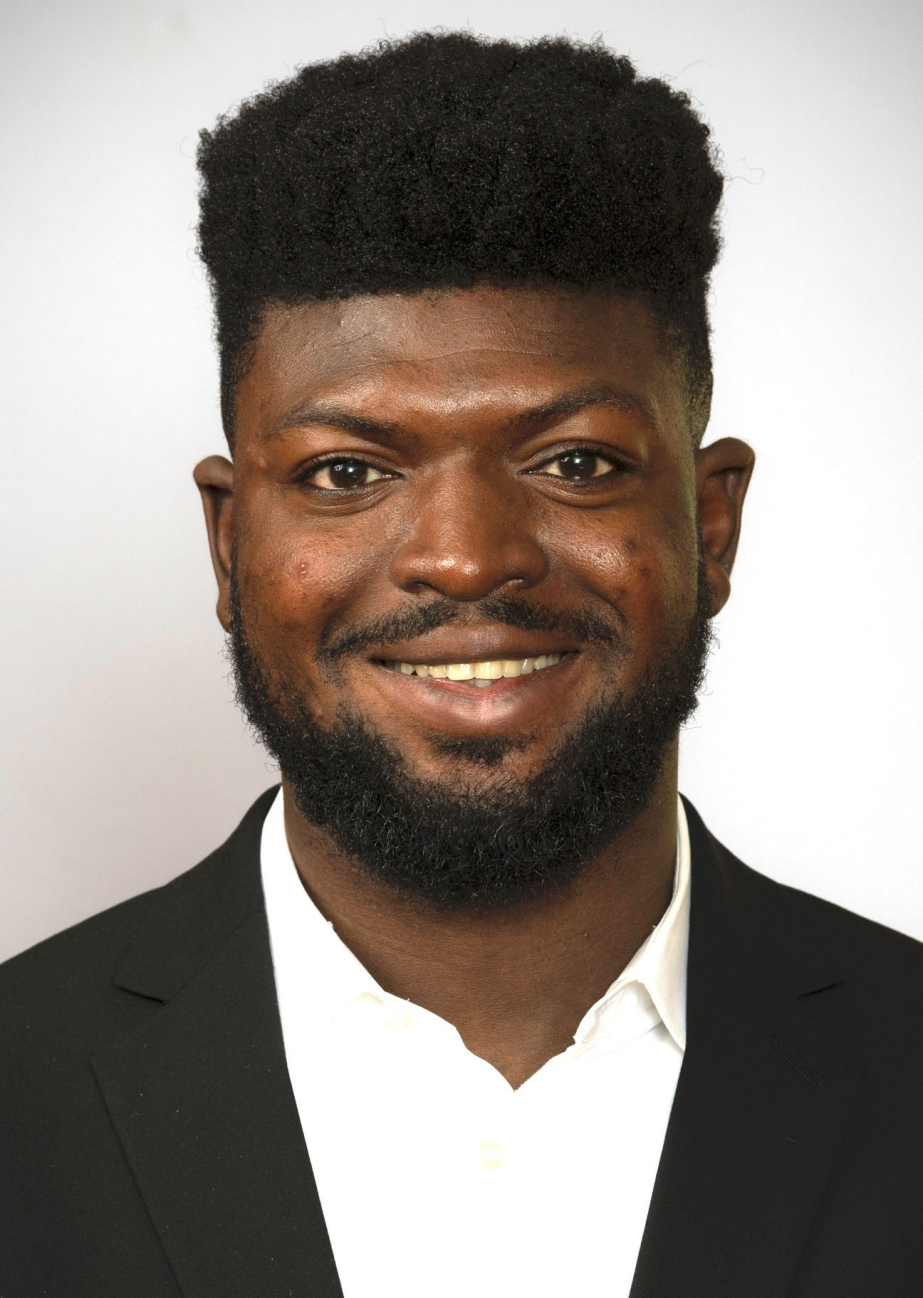

## Biographical Information

*Dominik Konkolewicz is a Professor and group leader in the Chemistry and Biochemistry Department at Miami University. The Konkolewicz group started in 2014, and consists of a team of 10 researchers. Projects in the Konkolewicz group include reprocessable and dynamic networks, responsive materials, light driven reactions, polymerization mechanisms and macromolecules that interact with biological molecules. A particular interest in the group is materials design through structure–property analysis, and tuning timescales of materials’ characteristics*.